# Clinical Guideline-Guided Outcome Consistency for Surgically Resected Stage III Non-Small Cell Lung Cancer: A Retrospective Study

**DOI:** 10.3390/cancers13112531

**Published:** 2021-05-21

**Authors:** Hiroaki Kuroda, Yusuke Sugita, Katsuhiro Masago, Yusuke Takahashi, Takeo Nakada, Eiichi Sasaki, Noriaki Sakakura, Rui Yamaguchi, Hirokazu Matsushita, Toyoaki Hida

**Affiliations:** 1 Department of Thoracic Surgery, Aichi Cancer Center Hospital, Nagoay 464-8681, Japan; y.takahashi@aichi-cc.jp (Y.T.); t.nakada@aichi-cc.jp (T.N.); nsakakura@aichi-cc.jp (N.S.); 2 Division of Oncoimmunology, Aichi Cancer Center Research Institute, Nagoya 464-8681, Japan; y.sugita07@gmail.com (Y.S.); h.matsushita@aichi-cc.jp (H.M.); 3 Department of Pathology and Molecular Diagnostics, Aichi Cancer Center, Nagoya 464-8681, Japan; masago@aichi-cc.jp (K.M.); esasaki@aichi-cc.jp (E.S.); 4 Division of Cancer Systems Biology, Aichi Cancer Center Research Institute, Nagoya 464-8681, Japan; r.yamaguchi@aichi-cc.jp; 5 Department of Thoracic Oncology, Aichi Cancer Center, Nagoya 464-8681, Japan; 107974@aichi-cc.jp

**Keywords:** clinical guideline, non-small cell lung cancer, outcome, overall survival, adjuvant chemotherapy, epidermal growth factor receptor, anaplastic lymphoma receptor tyrosine kinase

## Abstract

**Simple Summary:**

Evidence-based guidelines provide valuable management recommendations that can significantly improve patient treatment and outcome, thereby reducing clinical variability. Recent clinical trials demonstrated that personalised treatments based on genomic and immune profiles can contribute to the prognosis of non-small cell lung cancer (NSCLC). This retrospective study investigated whether guideline-consistency, including adjuvant treatments after surgical resection (ATSR) and guideline-matched first-line treatment for recurrence (GMT-R), could influence overall survival (OS). From 2006 to 2017, 308 patients with pathological stage III NSCLC were eligible, among whom 207 (67.2%) recurrence cases were identified. ATSR and GMT-R were allowed in 164 (53.2%) and 129 (62.3%) cases, respectively. The 5-year OS in guideline-consistent cases receiving ATSR and GMT-R was significantly better than that in guideline-inconsistent cases (*p* < 0.01). Subgroup analyses further revealed that the 5-year OS after propensity adjustment was significantly better in guideline-consistent than in guideline-inconsistent cases (*p* < 0.01). Hence, guideline-consistent treatment alternatives effectively contribute to better outcomes.

**Abstract:**

Clinical guidelines can help reduce the use of inappropriate therapeutics due to localism and individual clinician perspectives. Nevertheless, despite the intention of clinical guidelines to achieve survival benefit or desirable outcomes, they cannot ensure a robust outcome. This retrospective study aimed to investigate whether guideline-consistency, including adjuvant treatments after surgical resection (ATSR) and guideline-matched first-line treatment for recurrence (GMT-R), according to the genomic profiles and immune status, could influence overall survival (OS). From 2006 to 2017, the clinical data of 308 patients with stage III non-small cell lung cancer (NSCLC) after surgical resection were evaluated. ATSR and GMT-R were allowed in 164 (53.2%) and 129 (62.3%) patients cases after surgical pulmonary resection, among which 207 (67.2%) recurrences were identified. The 5-year OS in guideline-consistent cases was significantly better than that in guideline-inconsistent cases (*p* < 0.01). Subgroup analyses further showed that the 5-year OS after propensity adjustment was significantly better in guideline-consistent than in guideline-inconsistent cases (*p* < 0.01), but not in either ATSR or GMT-R (*p* = 0.24). These data suggest that the guideline-consistent alternatives, which comprise ATSR or GMT-R, can contribute to survival benefits in pathological stage III NSCLC. However, only either ATSR or GMT-R has a potential survival benefit in these patients.

## 1. Introduction

The landscape of anticancer agents available in the clinical setting has significantly evolved over the past 50 years. In particular, the combination of chemotherapy with cisplatin to target non-small cell lung cancer (NSCLC) that has progressed has allowed the support of a mean length of life of 8–12 months. However, the outcome of these patients still remains poor and further therapeutic options are limited [1]. In the 21st century, due to the bright results of molecular targeted techniques, genome medicine, and immunobiology, the diagnostic and therapeutic efficacy for NSCLC has greatly progressed. These advances have paved the way for physicians and surgeons to be realistically able to see results from translational research. Therefore, a strategy to unify fragmented treatment and, thereby, improve treatment efficacy in clinical practice is indispensable.

The clinical evidence-based quality of treatment indicators for NSCLC is clinically required to ensure adequate management and better treatment strategies. The treatment of NSCLC at various stages is established by correct clinical staging, and treatment strategies are delineated by multidisciplinary teams. Therefore, evidence-based clinical guidelines can provide physicians and surgeons with the same basic principles for conducting lung cancer treatment. Various clinical practice guidelines have been developed to reduce inappropriate treatments, eliminate local and geographic deviations, and authorise the effective use of cancer treatment resources. If resistance to guideline-based first- or second-line treatments is quickly developed, physicians may suggest additional therapies or best supportive care to patients harbouring a more advanced stage.

Our previous report on 2756 NSCLC patients whose tumours were surgically resected between 1990 and 2012 revealed that the 5-year overall survival (OS) rates were 47.6% and 24.1% for patients with stage IIIA (*n* = 536, 19%) and IIIB (+IIIC) (*n* = 146, 5%) cancer, which mainly had lymph node metastasis or involvement of neighbouring structures, respectively [2]. Staged-III NSCLC has locally advanced non-metastatic assets as well as a heterogeneous profile. Accurate staging of patients being investigated by multidisciplinary teams can pave the way for most effective treatments, such as neoadjuvant or adjuvant chemotherapy, with or without surgery, chemotherapy, or additional radiotherapy. Alternatively, for patients with resectable tumours, multimodality treatment, including surgery, can be offered in an attempt to improve survival. Adjuvant chemotherapy has been approved for the treatment of surgically resected stage IB–IIIA NSCLC and is recommended as a standard treatment strategy according to various guidelines [3].

Clinical guidelines have the intention to promote survival benefit or desirable outcomes based on selected randomised studies; however, they cannot ensure a robust outcome. This study focused on two main key words in perioperative clinical guidelines: ‘adjuvant treatments after surgically resection’ (ATSR) and ‘guideline-matched first-line treatment for recurrence’ (GMT-R). The aim of this study was to explore whether guideline-consistency could provide specific outcomes according to these two therapeutic alternatives.

## 2. Materials and Methods

In this prospective cohort, we examined 308 patients with staged-III primary NSCLC who underwent pulmonary resection at the Aichi Cancer Hospital between January 2006 and December 2017. This study was conducted in accordance with the Declaration of Helsinki. The institutional review board of the Aichi Cancer Centre approved this study (2020-1-614). Informed consent obtained by individuals was waived because of the retrospective nature of this cohort. The following exclusion criteria were applied: (1) salvage surgery; (2) patients having final diagnosis as small cell lung cancer or carcinoid; (3) induction chemotherapy with or without radiotherapy; and (4) sublobar resection. All patients with NSCLC underwent lobectomy or more with mediastinal lymph node dissection or sampling. Data postoperatively collected from patient records included age, gender, era, clinical N stage determined by positron emission tomography and computed tomography, prognostic nutrition index or PNI (calculated using the following formula = serum albumin levels (g/dL) × 10 + total lymphocyte count (per mm^3^) × 0.005)] [4], and smoking status (pack-years). Computed tomography is routinely used as the standard for preoperative lymph node staging, and the commonly used criterion for a clinical diagnosis of N evaluation is a short axis diameter > 10 mm. Resectable indication of cN2 is only single station. If multiple station metastases is clinically suspected, we performed endobronchial ultrasound-guided transbronchial needle aspiration. Pathological stages were defined according to the 8th edition of Union for the International Cancer Control (UICC)/American Joint Committee on Cancer (AJCC) TNM staging criteria [5].

The NCCN guideline [6] indicated these following recommendation: (a) The overall plan of treatment as well as needed imaging studies should be determined before any non-emergency treatment is initiated; (b) Anatomic pulmonary resection is preferred for the majority of patients with NSCLC. (c) N1 or N2 node resection and mapping should be a routine component of lung cancer resections—a minimum of three N2 stations sampled or complete lymph node dissection; (d) Patients with pathologic stage II or greater should be referred to medical oncology for evaluation; (e) The presence of N2-positive lymph nodes substantially increases the likelihood of positive N3 lymph nodes. Pathologic evaluation of the mediastinum must include evaluation of subcarinal station and contralateral lymph nodes; (f) Neoadjuvant chemotherapy would be considered, followed by surgery, when a patient is likely, based on initial evaluation, to require a pnumonectomy. According to guidelines, neoadjuvant treatment is recommended for cN2 disease, but we excluded the patients who had received neoadjuvant chemotherapy or chemoradiotherapy. Our institutional criteria for neoadjuvant therapy is mostly to escape the pnumonectomy. Therefore, we did not consider that enough evaluation of mediastinal lymph node was obtained, preoperatively.

### Statistical Analyses

All computations relied on standard software (SPSS version25.0; SPSS Inc, Chicago, IL, USA). Comparisons between the two groups were performed by Mann–Whitney *U*-tests. Propensity adjustment is defined as the conditional probability calculated by preoperative covariates. Propensity adjustment was estimated using a logistic model including limited variables, which showed a significant difference (*p* < 0.05) by univariate analyses. The Kaplan–Meier method was used to analyse survival rates in the patient subsets; between-group differences in survival were assessed with the log-rank test. Potential correlates of survival were subjected to univariate and multivariate analyses using the Cox proportional hazards regression model.

## 3. Results

### 3.1. Patient Flow Algorism

**Figure 1:**
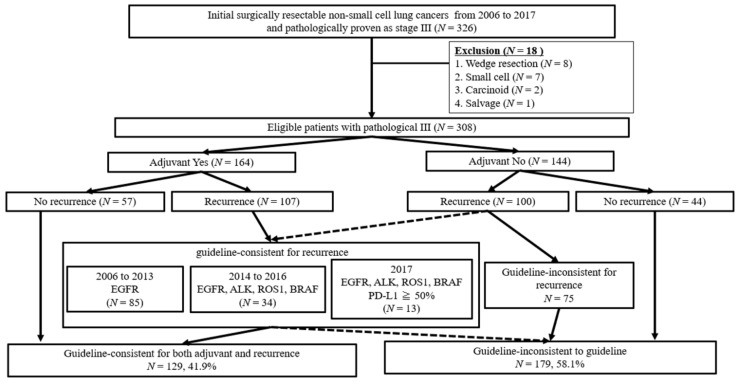
Patient flow diagram.

Between January 2006 and December 2017, 308 patients with surgically resected NSCLC were diagnosed with pN2 (cancer spread to 1–4 lymph nodes). Patients who received neoadjuvant chemotherapy were excluded from the analysis because precise information on lymph node mapping could not be obtained. *EGFR* mutations (exons 18–21) have been assessed using the cycleave PCR method since 2006. *ALK* rearrangement and *ROS1* were first screened by immunochemistry, and the final definition was performed by fluorescence in situ hybridisation. Information on these fusion genes has been clinically used since 2007 and 2016. *BRAF* assessment (exons 11 to 15) was based on reverse transcription PCR, coupled with direct sequencing, as previously reported [7]. The expression status of the programmed death-ligand 1 (PD-L1) was determined by immunostaining using two antibodies, either 28-8 or 22C3 pharmDx kits (Dako North America, Carpinteria, CA, USA), and the total proportion score was calculated. Patient flow diagram of this study is shown in Figure 1. The cases were classified as guideline inconsistent or consistent based on the National Comprehensive Cancer Network (NCCN) guideline for NSCLC. Overall, 179 guideline-inconsistent and 129 guideline-inconsistent cases were identified.

### 3.2. Patient Characteristics

Table 1 shows the relevant patient characteristics. According to clinical guidelines for recurrence, ATSR was established as follows: the molecular target drug for EGFR from 2006, for ALK from 2007, for BRAF from 2014, for ROS1 from 2016, for an immune checkpoint inhibitor from 2017, for tumour proportion score (TMS) ≥ 50% of program cell death protein 1 (PD-1). Guideline inconsistency was defined as patients with ATSR and GMT-R.

The methods for the analysis of each mutations, *EGFR*, *ALK*, *BRAF*, and *ROS1* have been previously described [8]. *EGFR* (exons 18–21) mutations were identified using the cycleave polymerase chain reaction method. *BRAF* (exons 11–15) mutation was assessed using fragment analysis, and the results were validated by direct sequencing. *ALK* and *ROS1* mutations were first screened using immunohistochemistry, and the final confirmation was performed using fluorescence in situ hybridization.

The guideline-inconsistent group (*n* = 179) comprised older patients (*p* < 0.01) and patients with lower prognostic nutritional index (*p* < 0.01) compared with guideline-consistent cases (*n* = 128). Patients within the guideline-consistent group were less likely to undergo lobectomy (*p* = 0.02) and were more like to have non-adenocarcinoma (*p* = 0.05). ATSR was performed in 35 (19.6%) guideline-inconsistent cases.

### 3.3. Surgical Outcomes and Therapeutic Efficacy in Recurred Patients

**Figure 2:**
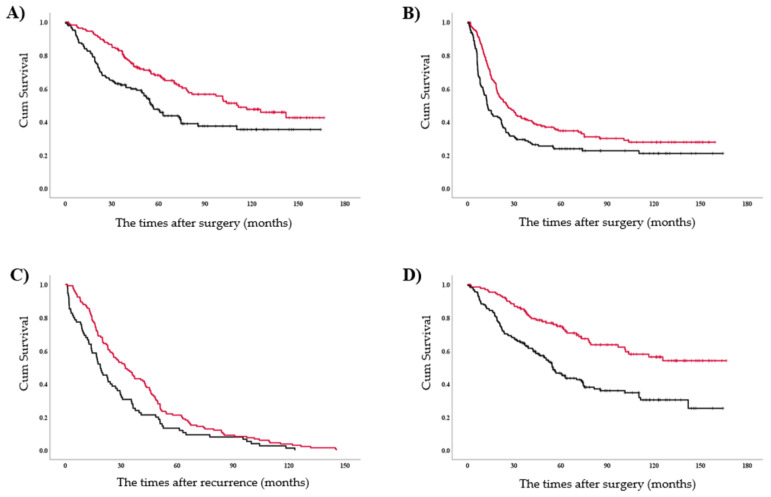
Kaplan–Meier curves. (**A**) Overall survival curve and (**B**) disease-free survival curve after surgical tumour resection stratified according to the adjuvant treatments. Red and black lines represent with and without adjuvant treatments, respectively. (**C**) Overall survival curve after recurrence stratified according to the guideline matched first-line treatment for recurrence. Red and black lines represent yes and no, respectively. (**D**) Overall survival after surgical tumour resection. Red and black lines represent guideline-consistent and guideline-inconsistent cases, respectively.

The median follow-up duration was 54.4 months (interquartile range (IQR): 30.1–92.5). The 5-year and median OS were significantly better in stage III cases who received ATSR (*n* = 164; 68.0% and 111.3 months, respectively) than in those who did not (*n* = 144; 47.6% and 56.0 months, respectively) (*p* < 0.01) (Figure 2a). Moreover, the 5-year and median disease-free survival (DFS) were significantly better in stage III patients who received ATSR (34.6% and 25 months, respectively) than in those who did not (*n* = 23.8% and 12.8%, respectively; *p* = 0.02) (Figure 2b).

Overall, 207 patients (67.2%) experienced tumour recurrence during the study period. As shown in Appendix A, the frequent mutation was EGFR (*n* = 96, 46.4%), followed by ALK (*n* = 8, 3.8%) and ROS1 (*n* = 1, 0.5%), while total proportion score ≥ 50% were seen in 5 (2.4%). Among them, target therapy was performed in 69 (71.8%) of EGFR, in 5 (62.5%) of ALK, and in 1 (100%) of ROS1, while 3 patients (60.0%) received immunecheck point inhibitor as first-line treatment. Seventy-two patients from ATSR (67.2%) were subjected to GMT-R, including local therapy in 6 (8.3%), chemotherapy only in 16 (22.2%), chemoradiotherapy in 5 (7.0%), and targeted therapy in 45 (62.5%). The 5-year and median OS were significantly better in recurred patients who received GMT-R (*n* = 132; 21.2% and 32.1 months, respectively) than in those who did not (*n* = 75; 13.3% and 18.8 months, respectively; *p* < 0.01) (Figure 2c). Furthermore, the 5-year and median OS were significantly better in the guideline-consistent group (*n* = 129; 74.8% and not reached, respectively) than in the guideline-inconsistent group (*n* = 179; 46.5% and 54.9%, respectively; *p* < 0.01) (Figure 2d).

### 3.4. To Investigate the Prognostic Factor for OS

Multivariate Cox regression analysis of OS after surgical tumour resection was performed according to the results of the univariate analysis. Univariate analyses revealed that age, male sex, prognostic nutritional index (<50), era (2006–2013), guideline-inconsistency, and any genetic mutations were independent OS predictors (Table 2). Multivariate analyses further confirmed that age, era (2006–2013), and guideline inconsistency were independent predictors (Table 2).

### 3.5. Subgroup Analyses for OS

**Figure 3:**
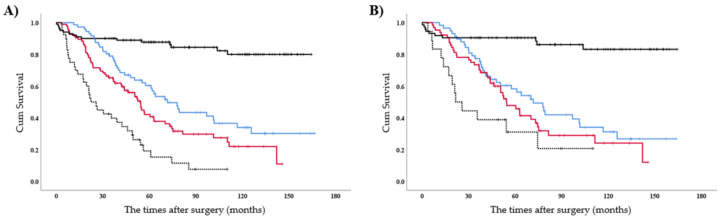
Kaplan–Meier curves in subgroup analyses. Overall survival curve after surgical tumour resection (**A**) before and (**B**) after propensity adjustment. Black, blue, red, and dotted lines represent no recurrence (NR group), both adjuvant treatments after surgical resection and guideline-matched first-line treatment for recurrence (guideline-consistent group), either treatment (EAG group), and neither treatment (guideline-inconsistent group).

The study cohort was divided in four groups, as follows: no recurrence (NR), guideline-consistent, either ATSR or GMT-R (EAG), and guideline-inconsistent. The 5-year OS in the guideline-consistent group was significantly better than that in the EAG (*p* = 0.03) and guideline-inconsistent groups (*p* < 0.01). Nonetheless, the 5-year OS in the EAG groups was significantly better than that in the guideline-inconsistent group (*p* < 0.01; Figure 3a).

Propensity adjustment was estimated using a logistic model including age, sex, era, pack-year, prognostic nutritional index, and any mutations, which were selected based on the results of univariate analyses (Table 3). The 5-year OS after propensity adjustment in the guideline-consistent group was significantly better than that in the guideline-inconsistent cases (*p* < 0.01), but not in the EAG group (*p* = 0.24; Figure 3b). However, a significant difference was not observed in the 5-year OS after propensity adjustment between the EAG and guideline-inconsistent groups (*p* = 0.09; Figure 3b).

## 4. Discussion

According to the clinical guidelines, complete dissection of at least three mediastinal nodal stations is recommended for the treatment of NSCLC. After complete pulmonary resection with pN2 proven and negative margins, adjuvant chemotherapy is recommended, whereas for incomplete or complete unknown cases either re-resection or additional chemotherapy or radiotherapy is recommended. In clinical practice, therapeutic guidelines for advanced NSCLC can be substituted by those for metastatic NSCLC. This study was designed to explore whether adherence to therapeutic management guidelines could provide survival benefit for patients with stage III NSCLC. Multimodality staging may have led to superior patient outcomes by supporting more accurate staging and, subsequently, more appropriate treatment allocation. Nevertheless, one clinical question remains, “which of these two possibilities (adjuvant chemotherapy or therapeutic adherence) has a greater impact for metastatic NSCLC?” Herein, adherence to clinical guidelines for both ATSR and GMT-R showed promising potential to improve patient survival.

During the last decade, the development of molecular targets has dramatically evolved, enabling precision medicine and personalised treatment alternatives. The six currently approved U.S. Food and Drug Administration (FDA) EGFR inhibitors have demonstrated excellent efficacy regarding objective response rate and prognosis in EGFR-positive NSCLC, with fewer adverse effects [9,10]. Erlotinib was first approved in 2013 by the FDA as a first-line treatment, and afatinib was approved later on in the same year. In the present study, analysis of *EGFR* in all stage III NSCLC patients showed that 39.9% (123/308) harboured *EGFR* mutations. These patients were authorised to receive EGFR inhibitors as first-line treatment for tumour recurrence, in agreement with the guidelines. From 2006 to 2013, 92 (44.4%) patients were diagnosed with metastatic NSCLC, among whom 32 (34.8%) harboured *EGFR* mutations. First-line EGFR inhibitors were clinically used in 11 (34.4%) of these patients after approval by the institutional review board.

*ALK* rearrangement is widely recognized as being associated with NSCLC at younger age, never-to-light smoking, and a preference to affect the central nervous system, which contributes to a dismal prognosis [11]. Crizotinib was first approved by the FDA for metastatic NSCLC in 2011 [12]. Moreover, the ALFEX trial comprising 303 Asian advanced NSCLC patients harbouring the *ALK* rearrangement revealed a clinical benefit of alectinib as a first-line treatment [13]. In the present cohort of patients with surgically resected NSCLC from 2007 to 2012, *ALK* was assessed in 68.7% (136/198) of patients, among whom 0.6% (9/136) harboured an *ALK* rearrangement. In addition, our previous report revealed a significantly higher incidence of occult lymph node metastases in *ALK*-positive NSCLC, which makes these patients good candidates for adjuvant chemotherapy according to the clinical guidelines [14].

The *BRAF* and *ROS1* status in this cohort have been investigated since 2014 and 2016, respectively, but the BRAF inhibitors dabrafenib and trametinib were only approved by the FDA in 2017. No *BRAF*-positive patients were identified in this study, whereas 2.6% (1/43) of patients with stage III NSCLC were *ROS1*-positive; thus, crizotinib was used as per the guidelines as a first-line treatment for tumour recurrence.

Recently, immune checkpoint inhibitors (ICIs) have dramatically revolutionised the treatment of metastatic or advanced NSCLC, but their efficacy is limited to a well-equipped immune microenvironment [6]. Pembrolizumab was clinically approved in 2016 as a first-line treatment for metastatic NSCLC in patients with a total proportion score ≥ 50% and without *EGFR* or *ALK* mutations after the KEYNOTE 024 and 042 clinical trials [15,16]. In agreement, our previous study also suggested that ICI treatment was significantly less efficacious in patients with *ALK* rearrangement than in patients with *EGFR* mutations, and that PD-L1 expression was not a critical biomarker for ICI treatment in patients with one of these mutations [8]. Herein, six patients with recurrence (20.0%, 6/30) were treated with first-line ICI, according to the clinical guidelines stipulated since 2016.

Wilshire et al. reported that guideline-inconsistent diagnosis and staging occurred in 58% of clinical stage III cases, which was associated with incomplete staging, a higher number of additional procedures, and delayed management [17]. Moreover, absence of invasive mediastinal lymph node sampling in 43% of patients suspected of having clinical stage III disease before the initiation of treatment was associated with a higher number of additional procedures and delayed management [17]. In the present study, pathologically proven N2 cases were specifically selected, which may have contributed to obtaining precise efficacy in treatments after surgical resection. In addition, several prospective randomised trials in patients with stage I-IIIA NSCLC have demonstrated the survival efficacy of cisplatin-based adjuvant chemotherapy [18,19].

Herein, the single centre clinical data from before the establishment of various clinical guidelines were evaluated. Mutational information from operative specimens were assessed using direct sequencing, which allowed determination of the therapeutic statistics according to the mutational status of the patients, which also reflects the social changes over time. ATSR was established as a survival benefit of ~11% in DFS, but an additional benefit of 20% was identified in OS. Hence, guideline inconsistency, even in pathological stage III, might improve the survival outcome and allow application of precision medicine by introducing the new strategies established from newly acquired knowledge.

This study has several limitations. First, the data were collected and analysed retrospectively, which could have caused selection bias. Second, this study was based on data collected at a single centre with a relatively middle scale. Third, direct sequencing is not currently performed as a standard clinical tool because it only investigates a limited gene sequence portion. In addition, it should be also noted that the systematic process for identifying genomic mutations only recently was made available; for example, *ALK* since 2007, *BRAF* since 2014, and *ROS1* since 2016. Therefore, only few patients included in the present analysis were treated with more specific treatments. Nevertheless, targeted selection or exclusion of these patients did not seem reasonable as they would not represent the typical phases of medical application development or ongoing clinical investigation. Fourth, we restricted the therapeutic alternative to first-line treatment only.

## 5. Conclusions

This retrospective study suggests that a guideline-consistent treatment alternative comprising ATCR and GMT-R, depending on the genomic profiles and immune environments, can provide a survival benefit for patients with pathological stage III NSCLC. Both ATCR and GMT-R are optional in clinical practice, but at least one of them may be recommended to improve the outcome of these patients.

## Figures and Tables

**Table 1:** Clinicopathological characteristics before propensity adjustment.

Characteristics		Inconsistent	Consistent	*p*-Value
		*n* = 179	*n* = 129	
Age (years old), median		67	63	<0.01
	IQR	(61–73)	(58–67)	
Gender, male (%)		104 (58.1%)	73 (56.6%)	0.79
Era				0.82
	2006–2013	116 (64.8%)	82 (63.6%)	
	2014–2017	63 (35.2%)	47 (36.4%)	
Smoking history (pack-year), median		34.0	16.0	0.08
	IQR	(0–52.0)	(0–46.5)	
Prognostic nutritional index, median		49.8	52.4	<0.01
	IQR	(46.3–53.3)	(49.3–54.7)	
Clinical stage N (number, %)				0.31
	cN0	98 (54.7%)	63 (48.8%)	
	cN1–2	81 (45.3%)	66 (51.2%)	
Clinical stage				0.80
	cI	68 (38.0%)	51 (39.5%)	
	cII	46 (25.7%)	32 (24.8%)	
	cIII	65 (36.3%)	46 (35.7%)	
Histology (number, %)				0.05
	Adenocarcinoma	117 (65.4%)	98 (76.0%)	
	Others	62 (34.6%)	31 (24.0%)	
Type of procedures (number, %)				0.02
	Lobectomy	149 (83.2%)	119 (92.2%)	
	Pneumonectomy/Bilobectomy	30 (16.8%)	10 (7.8%)	
ATCR (yes, %)		35 (19.6%)	129 (100%)	<0.01
Pathological-Stage				<0.01
	IIIA	167 (93.2%)	123 (95.3%)	
	IIIB	12 (6.7%)	6 (4.7%)	
Single lymph node involvement				0.35
	(yes, %)	29 (16.2%)	16 (12.4%)	
Mutation status (yes/no/uninformative)				
	*EGFR*	68/111/0	55/74/0	0.41
	*ALK*	5/140/34	6/95/28	0.35
	*BRAF*	0/62/117	0/40/89	NA
	*ROS1*	0/24/155	1/16/107	NA
Treatment after recurrence (yes, %)		136 (76.0%)	74 (57.4%)	0.04
	Local control	26 (19.1%)	7 (9.0%)	
	Chemotherapy ± Radiotherapy	55 (40.4%)	21 (28.4%)	
	Molecular target drug	37 (27.2%)	44 (59.5%)	
	Immune checkpoint inhibitor	4 (3.0%)	2 (2.7%)	
	Others	14 (10.3%)	0 (0%)	

ALK, anaplastic lymphoma kinase; ATSR, adjuvant treatments after surgical resection; BRAF, v-raf murine sarcoma viral oncogene homolog B1; EGFR, epidermal growth factor receptor; IQR, interquartile range; NA, not available; ROS1, c-ros oncogene 1.

**Table 2:** Univariate and multivariate analyses of overall survival.

Variables		Univariate	Multivariate	
		*p*-Value	Hazard Ratio (95% CI ^1^)	*p*-Value
Patient characteristics				
	Age	<0.01 *	1.02 (1.01–1.04)	0.02 *
	Male	<0.01 *	0.73 (0.50–1.06)	0.10
	Pack-year	0.11		
Prognostic nutritional index				
	Score < 50	0.02 *	0.74 (0.54–1.03)	0.08
Era				
	2006–2013	<0.01 *	0.50 (0.33–0.76)	<0.01 *
Clinical N stage				
	N1–2	0.27		
Histology				
	Adenocarcinoma	0.57		
Procedures				
	More than lobectomy	0.81		
Guideline				
	Inconsistent	<0.01 *	0.49 (0.34–0.71)	<0.01 *
Any mutation				
	Yes	<0.01 *	1.36 (0.94–1.97)	0.10
Pathological N status				
	Single involvement	0.39		

* Statistically significant *p*-value. ^1^ CI, confidential index.

**Table 3:** Clinicopathological characteristics after propensity adjustment.

Characteristics		Inconsistent	Consistent	*p*-Value
		*n* = 106	*n* = 108	
Age (years old), median		64	64	0.98
	IQR	(60–68)	(58–69)	
Gender, male (%)		61 (57.5%)	59 (54.6%)	0.67
Era				0.97
	2006–2013	69 (65.1%)	70 (64.8%)	
	2014–2017	37 (34.9%)	38 (35.2%)	
Smoking history (pack-year), median		20.0	32	0.49
	IQR	(0–49.1)	(0–50.8)	
Prognostic nutritional index, median				0.81
	IQR	36 (34.0%)	35 (32.4%)	
Any mutation (*EGFR*/*ALK*/*BRAF*/*ROS1*)				
	(yes, %)	50 (47.2)	50 (46.3%)	0.90

ALK, anaplastic lymphoma kinase; ATSR, adjuvant treatments after surgical resection; BRAF, v-raf murine sarcoma viral oncogene homolog B1; EGFR, epidermal growth factor receptor; IQR, interquartile range; NA, not available; ROS1, c-ros oncogene 1.

**Figure A1:**
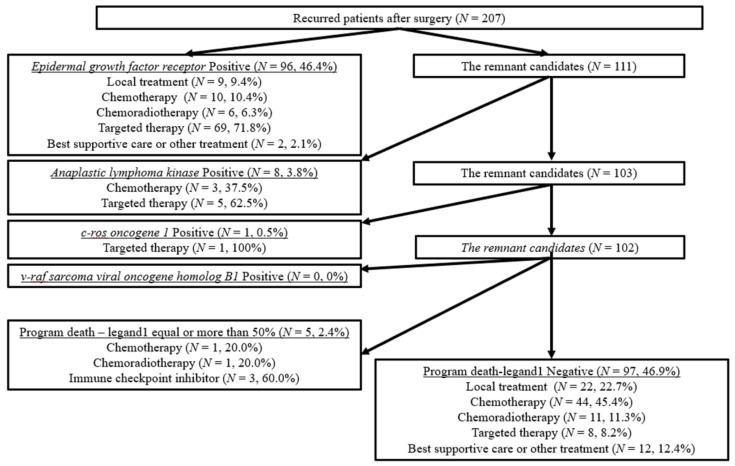
Patient flow diagram after recurrence according to the mutational information and program death—ligand 1 status.

## Data Availability

The data presented in this study are available in this article.
